# Yttrium-90 radioembolization as a possible new treatment for brain cancer: proof of concept and safety analysis in a canine model

**DOI:** 10.1186/s13550-020-00679-1

**Published:** 2020-08-17

**Authors:** Alexander S. Pasciak, Sasicha Manupipatpong, Ferdinand K. Hui, Larry Gainsburg, Rebecca Krimins, M. Christine Zink, Cory F. Brayton, Meaghan Morris, Jaime Sage, Danielle R. Donahue, Matthew R. Dreher, Dara L. Kraitchman, Clifford R. Weiss

**Affiliations:** 1 School of Medicine, The Johns Hopkins University School of Medicine, 1800 Orleans St, Baltimore, MD 21287 USA; 2 Department of Radiology and Radiological Science, Division of Vascular and Interventional Radiology, The Johns Hopkins University School of Medicine, Baltimore, MD USA; 3 Mid-Atlantic Veterinary Neurology and Neurosurgery, Baltimore, MD USA; 4 Department of Molecular and Comparative Pathobiology, The Johns Hopkins University, Baltimore, MD USA; 5 Department of Radiology and Radiological Science, Express Radiology Research Lab, The Johns Hopkins University School of Medicine, Baltimore, MD USA; 6 Department of Radiology and Radiological Science, Veterinary Clinical Trials Network, The Johns Hopkins University School of Medicine, Baltimore, MD USA; 7 Department of Anesthesiology and Critical Care Medicine, The Johns Hopkins University School of Medicine, Baltimore, MD USA; 8 Department of Pathology, The Johns Hopkins University School of Medicine, Baltimore, MD USA; 9 MRI Vets, PLLC, Georgetown, TX USA; 10 Mouse Imaging Facility, National Institutes of Health, Bethesda, MD USA; 11 Biocompatibles UK Ltd., a BTG International group company, Farnham, Surrey, UK; 12 Department of Radiology and Radiological Science, Center for Image-Guided Animal Therapy, The Johns Hopkins University School of Medicine, Baltimore, MD USA; 13 Department Biomedical Engineering, The Johns Hopkins Whiting School of Engineering, Baltimore, MD USA

**Keywords:** Y90, SIRT, Glioma, Radiation therapy, Brain cancer, Canine model

## Abstract

**Purpose:**

To evaluate the safety, feasibility, and preliminary efficacy of yttrium-90 (^90^Y) radioembolization (RE) as a minimally invasive treatment in a canine model with presumed spontaneous brain cancers.

**Materials:**

Three healthy research dogs (R1–R3) and five patient dogs with spontaneous intra-axial brain masses (P1–P5) underwent cerebral artery RE with ^90^Y glass microspheres (TheraSphere). ^90^Y-RE was performed on research dogs from the unilateral internal carotid artery (ICA), middle cerebral artery (MCA), and posterior cerebral artery (PCA) while animals with brain masses were treated from the ICA. Post-treatment ^90^Y PET/CT was performed along with serial neurological exams by a veterinary neurologist. One month after treatment, research dogs were euthanized and the brains were extracted and sent for microdosimetric and histopathologic analyses. Patient dogs received post-treatment MRI at 1-, 3-, and 6-month intervals with long-term veterinary follow-up.

**Results:**

The average absorbed dose to treated tissue in R1–R3 was 14.0, 30.9, and 73.2 Gy, respectively, with maximum doses exceeding 1000 Gy. One month after treatment, research dog pathologic analysis revealed no evidence of cortical atrophy and rare foci consistent with chronic infarcts, e.g., < 2-mm diameter. Absorbed doses to masses in P1–P5 were 45.5, 57.6, 58.1, 45.4, and 64.1 Gy while the dose to uninvolved brain tissue was 15.4, 27.6, 19.2, 16.7, and 33.3 G, respectively. Among both research and patient animals, 6 developed acute neurologic deficits following treatment. However, in all surviving dogs, the deficits were transient resolving between 7 and 33 days post-therapy. At 1 month post-therapy, patient animals showed a 24–94% reduction in mass volume with partial response in P1, P3, and P4 at 6 months post-treatment. While P2 initially showed a response, by 5 months, the mass had advanced beyond pre-treatment size, and the dog was euthanized.

**Conclusion:**

This proof of concept demonstrates the technical feasibility and safety of ^90^Y-RE in dogs, while preliminary, initial data on the efficacy of ^90^Y-RE as a potential treatment for brain cancer is encouraging.

## Key points

Question: Can ^90^Y radioembolization be safely used for the intra-arterial treatment of axial brain cancers?

Pertinent findings: In this pre-clinical trial, cerebral artery delivery of ^90^Y radioembolization was successfully performed on both healthy research dogs and patient dogs with spontaneous intra-axial brain cancers. Absorbed doses ranging from 45.4 to 76.7 Gy were delivered to intra-axial lesions in patient dogs while healthy research dog models received doses up to 73.2 Gy to large volumes of brain tissue with no permanent neurological sequelae in either group.

Implications for patient care: While embolic trans-arterial therapies are typically considered to be contraindicated in neuro-oncology, radioembolization with ^90^Y glass microspheres may have a tolerable level of toxicity and warrants further investigation as a potential treatment for brain cancer.

## Introduction

Glioblastoma multiforme (GBM) is the most common and aggressive cancer of the central nervous system, with an age-adjusted incidence rate of 3.2 per 100,000 individuals [1] and a median survival time of 15 months [2]. The standard of care consists of surgical resection followed by adjuvant chemoradiotherapy with temozolomide [2]. Even with treatment, however, local recurrence of GBM within 2 cm of the original tumor margin is common due to diffuse cancer cell infiltration beyond imaged tumor boundaries [3–7]. Standard of care radiation therapy as a part of new-onset GBM treatment using external beam radiation therapy (EBRT) is typically prescribed with fractionated doses over 6 weeks for a total dose of 60 Gy to the tumor, a threshold at which survival benefits are maximal and neurotoxicity is minimal [8, 9]. Other radiation therapies include brachytherapy and stereotactic radiosurgery; however, both are associated with greater neurotoxicity and are typically reserved for relapsing GBM despite providing some benefits over EBRT [10–12].

Yttrium-90 (^90^Y) trans-arterial radioembolization (RE) has been used to treat human primary and metastatic liver cancer in the USA for over 20 years. Catheter-directed RE delivers high radiation doses, sometimes up to 1000 Gy thanks to the selective targeting of hypervascular tumors, compared to EBRT where absorbed dose rarely exceeds 70 Gy [13]. At the same time, RE minimizes non-target radiation with an average tissue penetration of 4.1 mm [14] as opposed to 3–4 cm in typical I-125 brachytherapy or EBRT where the entire cortex may be exposed to substantial doses of radiation [15–17]. Intra-arterial delivery of RE to intra-axial brain cancers raises the concern of ischemic changes resulting from the embolic effects of microspheres in normal brain tissue. The potential of RE to treat GBM will depend on the balance of embolic-related ischemic effects and the inherent ability to deliver high doses of radiation to hypervascular tumors while limiting normal tissue exposure.

In the design of this study, there was a significant thought put into proper device selection for ^90^Y cerebral artery RE. Currently, there are two FDA-approved ^90^Y microsphere devices in the USA, glass TheraSphere (Biocompatibles Ltd., UK) and resin SIRSpheres (SIRTeX Medical, Sydney, Australia). The TheraSphere product is advantageous for this potential application in intra-axial brain cancers because each microsphere contains up to 40× the radioactivity of other RE devices at the time of treatment [18]. This may allow for the delivery of radiation doses exceeding those typically in EBRT with fewer microspheres and a reduced embolic burden compared to the resin product.

In this proof of concept investigation, we explored the safety, feasibility, and preliminary efficacy of cerebral ^90^Y-RE using a healthy canine model and in canines with spontaneous intra-axial brain masses which were presumed neoplastic based on pre-treatment imaging.

## Methods

The Johns Hopkins Institutional Animal Care and Use Committee approved the animal protocol used in this study.

**Fig. 1:**
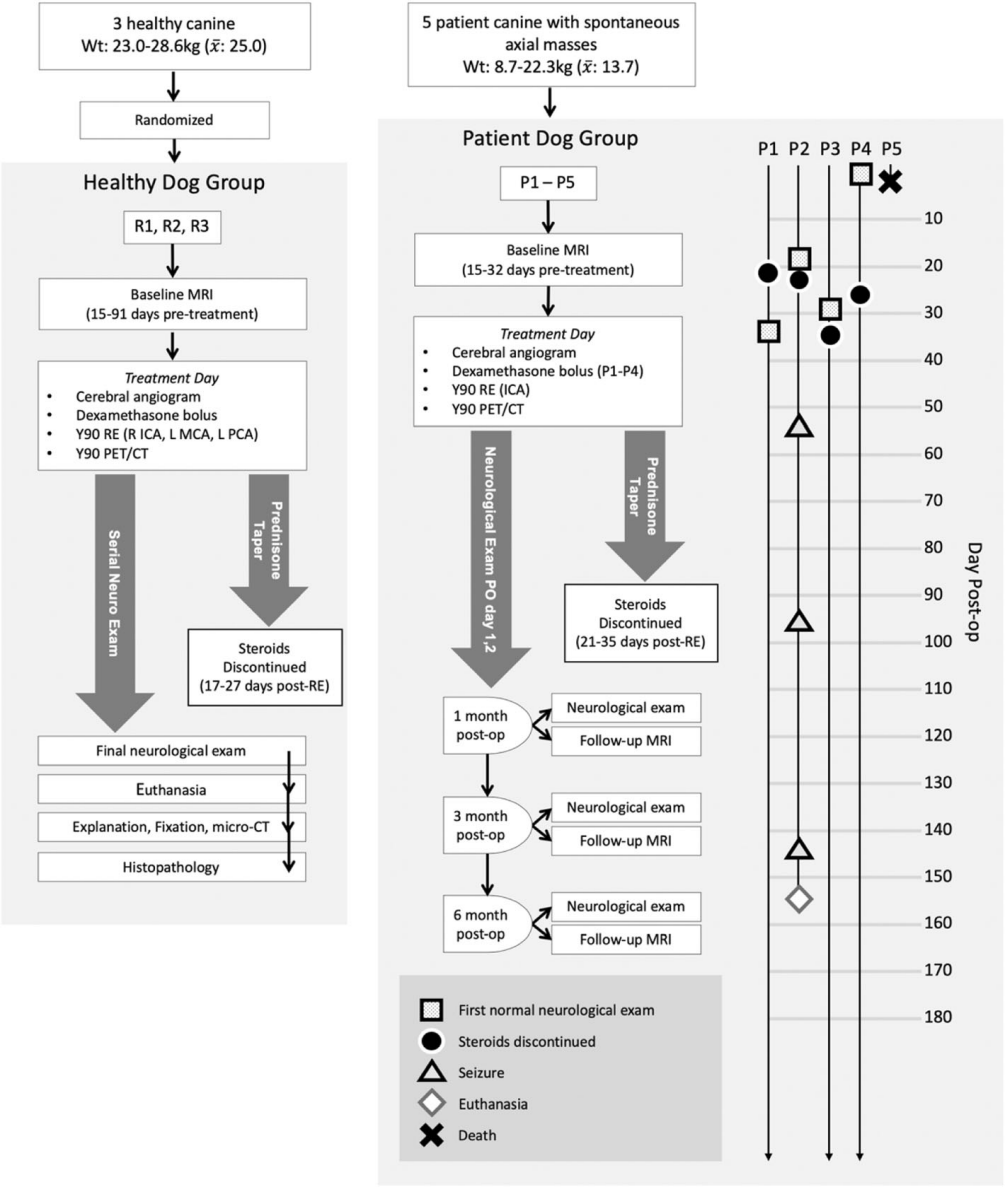
Flowchart of the clinical protocol used for cerebral ^90^Y-RE in research and patient dogs. An event plot is shown for patient animals as a function of days post-^90^Y-RE. All animals experienced generalized seizure activity prior to therapy (Table 1, Supplemental Material)

Three healthy research dogs (R1–R3) and five patient dogs with spontaneous intra-axial presumed neoplastic brain masses (P1–P5) underwent RE (Fig. 1) with Y-90 glass microspheres (Biocompatibles Ltd., UK).

### Inclusion and exclusion criterion for patient dogs

Patient dogs were at least 1 year of age with body weight greater than 4.4 kg and with MRI confirming the presence of an intra-axial lesion of at least 1 cm in size or greater. Priority was given to masses fed primarily by the branches of a single cerebral artery or in close proximity to the brainstem or cerebellum. Any prospective patients with a life expectancy of less than 2 weeks, bilateral tumor burden, previous or current oncological treatment of the tumor, or treatment with another investigational drug or intervention were ineligible for the study per our exclusion criteria. Extracranial malignancies or any evidence of serious systemic disorder or another condition that would preclude the evaluation of the safety and activity of the device were also grounds for exclusion from the trial. Inclusion of patient dogs was also contingent upon agreement by the team veterinarians, veterinary anesthesiologist, veterinary neurologist, interventional radiologists, and radiation physicist and informed consent of the owner.

### Treatment procedure (R1–R3, P1–P5)

An MRI for treatment planning was performed 15–32 days prior to treatment on P1–P5 as well as a baseline neurological examination performed by a veterinary neurologist (LG) on the day of treatment. Pre-procedural high-dose IV dexamethasone (0.1 mg/kg) was administered in all animals except P5. Cerebral angiography was performed under general anesthesia. Fentanyl, midazolam, and propofol administered intravenously were used for induction, and anesthesia was maintained with a 1–3% isoflurane inhalant and 100% oxygen after endotracheal tube placement. Fluoroscopically guided interventions were performed on a monoplane Siemens Artis Zee (Siemens Medical, Malvern, PA) C-arm by a team consisting of a board-certified neurointerventional radiologist (FH), board-certified interventional radiologist (CW), board-eligible veterinary anesthesiologist (RK), and veterinarians (DK, LG).

Ultrasound guidance was used to obtain percutaneous femoral access. Following placement of a 5-Fr sheath, a 5-Fr Impress hydrophilic vertebral catheter (Merit, South Jordan, UT) was advanced into the vertebral (R2–R3) or common carotid artery (R1, P1–P5) using a Bentson guidewire (Cook, Bloomington, IN). An Excelsior SL-10 microcatheter with Synchro 2 microwire (Stryker Neurovascular, Freemont, CA) was used to access the ICA (R1, P1–P5) or, via a vertebral approach, the PCA (R2) or MCA (R3). Intra-arterial nitroprusside was used on R1 as a spasmolytic. ^90^Y glass microspheres were delivered through the SL-10 using the FDA-approved TheraSphere delivery system (TheraSphere Administration Set and Accessory Kit) at a target infusion rate of 10 mL/min (half of the recommended 20 mL/min for the delivery system). Two complete flushes (40 mL total volume) were performed through the delivery system. Following RE, the catheters and sheath were removed and the pressure was held on the groin for hemostasis.

Immediately after treatment, a ^90^Y PET/CT (Biograph mCT, Siemens Medical, Knoxville, TN) was performed to document the technical success and for post-treatment dosimetry. Post-treatment PET/CT-based dosimetry was performed using the local deposition method [19]. ^90^Y PET/CT scan parameters have been previously described [18] and were 1-bed position at 20-min duration, resolution recovery, time-of-flight, ordered subset expectation maximization with 2 iterations and 21 subsets, allpass and 5-mm Gaussian reconstruction filter, and 512 × 512 matrix size. All dosimetry was performed using image sets reconstructed with the allpass filter.

### Treatment planning

Treatment of R1–R3 was performed in a dose-escalation manner with an administered activity of 0.0192, 0.0246, and 0.0161 GBq in R1–R3, respectively. Treatment planning was based on MR volumetry and estimated contours of the typical perfused region for PCA and MCA, as well as hemispherical volume for the treatment from the ICA. Using the MIRD schema [20], the expected single-compartment absorbed dose was 27.9, 66.5, and 73.0 Gy in R1–R3, respectively. R1–R3 were treated using ^90^Y glass microspheres 10 days post-calibration, i.e., on the 2nd week Wednesday. The lowest calibrated TheraSphere unit dosage available is 3.0 GBq, and at the device expiration (12 days post-calibration), this would equate to ~ 0.13 GBq. Therefore, the dosage was manually reduced using aseptic technique prior to the procedure to obtain the activity infused at the time of treatment (Table 1). Manual reduction of pre-calibration dose vials was performed on Thursday-Friday before the calibration using a conventional clinical dose calibrator after which the vial was allowed to decay to the aforementioned activity at treatment day.

**Table 1:** Treatment dose data for research dogs

		R1	R2	R3
	Infused activity (GBq)	0.019	0.025	0.016
	Treatment days post-calibration	10	10	10
	Volume of embolic (mL)*	0.0011	0.0015	0.00094
	Delivery	ICA	MCA	PCA
	Perfused fraction^†^	1	0.61	0.26
	Single compartment dose (Gy)	27.9	66.5	73
	Dosimetry method	MicroCT	MicroCT	MicroCT
Perfused territory	Mean dose (Gy)	14.0	30.9	73.2
	Max dose (Gy)	4484.0	10,231.0	8864.0
	V20	19.1	57.9	87.1
	V30	12.8	33.4	78.0
	V50	6.0	12.4	67.5
	V60	4.1	8.1	44.8
	V80	2.1	3.9	32.4
	V100	1.2	2.3	23.2
Basal ganglia	Mean dose (Gy)	55.9	63.4	18.3
	Max dose (Gy)	6738.0	10,231.0	5764.0
	V20	62.3	78.7	21.4
	V30	52.7	66.2	15.4
	V50	35.9	43.7	9.3
	V60	29.9	35.1	7.7
	V80	21.2	23.6	5.5
	V100	15.8	16.7	3.9
Hemisphere^‡^	Mean dose (Gy)	14.0	21.8	17.9
	Max dose (Gy)	4484.0	10,231.0	8864.0
	V20	19.1	39.1	18.5
	V30	12.8	22.6	16.0
	V50	6.0	8.4	12.3
	V60	4.1	5.4	10.6
	V80	2.1	2.7	7.7
	V100	1.2	1.6	5.5

*Calculated based on mean 27.5-μm sphere diameter and an ideal 2500 Bq/sphere specific activity at calibration

^†^Fraction of hemisphere perfused based on cone beam CT with the microcatheter at the treatment position

^‡^Embolized cortex (left or right) exclusive of brainstem and cerebellum

**Fig. 2:**
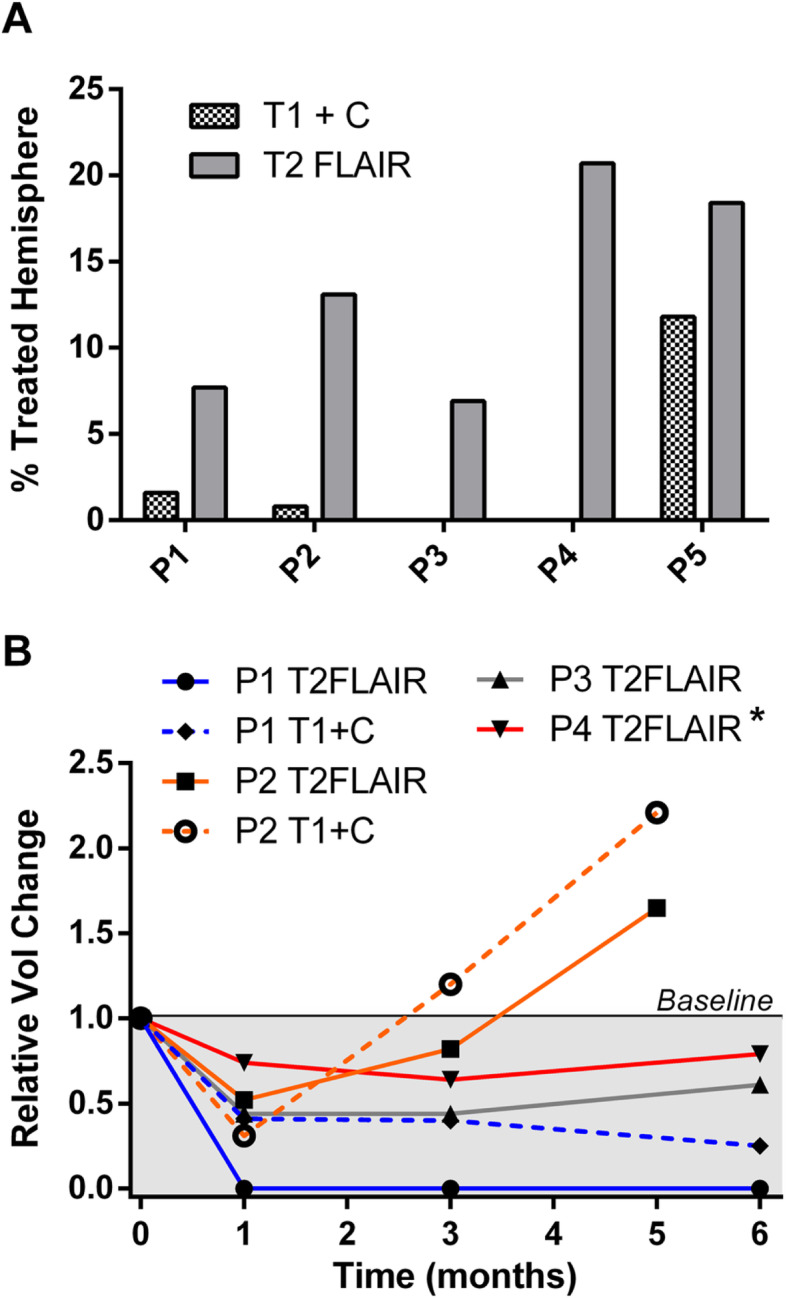
Intra-axial mass volumetry. **a** Pre-treatment volume as the percentage of the total treated hemisphere volume. **b** Relative change in volume of the mass as a function of time post-^90^Y-RE. *At 6 months, P4 developed new T1 post-contrast enhancement not noted at baseline; however, the overall volume of the mass was still smaller than pre-treatment

In P1–P5, pre-treatment MRI scans and hemispherical cortical volumes were used individually for treatment planning. Using the MIRD method, a treatment planning goal of 40 ± 10 Gy to the hemisphere was used for P1. Subsequent animals received a target of 50 ± 10 Gy unless the mass demonstrated a measurable contrast-enhancing component on post-contrast T1-weighted imaging, in which case a treatment target of 60 ± 10 Gy was applied. Space-occupying mass volume calculation from pre-treatment MRI was performed using MIPAV [Medical Image Processing, Analysis, and Visualization, National Institutes of Health, Bethesda, MD]. Figure 2a details the fraction of each hemisphere occupied by the mass based on T2 FLAIR or T1 post-contrast enhancing signal.

The infused ^90^Y activity on treatment day was 0.0183, 0.0478, 0.0351, 0.0414, and 0.0486 GBq in dogs P1–P5, respectively. Once again, these activities are far lower than what is commercially available. Therefore, manual reduction of TheraSphere unit dosages to obtain patient dog treatment day activities was performed using the same technique as described above for R1–R3. The associated average MIRD absorbed doses were 34.9, 63.7, 45.0, 45.2, and 62.2 Gy. Treatments occurred 11, 5, 10, 5, and 8 days post-calibration in P1–P5, respectively. Additional dosimetric and treatment planning details are provided in Tables 1 and 2.

**Table 2:** Treatment dose data for patient dogs with spontaneous intra-axial masses

		P1	P2	P3	P4	P5* (extended)	P5* (extended)	P5* (actual)
	First diagnosis (days before treatment)	16	35	35	33	28		
	Pre-treatment diagnosis	Vascular neoplasm vs vascular anomaly	Astrocytoma^†^	Low-grade glioma	Glial neoplasm	Astrocytoma^†^		
	Infused activity (GBq)	0.0183	0.0478	0.0351	0.0414	0.0486	–	–
	Treatment days post-calibration	11	5	10	5	8		
	Volume of embolic (mL)	0.0014	0.00076	0.0021	0.00066	0.0017		
	Pre-treatment mass vol (mL)	2.76	5.82	3.53	8.66	8.48	–	–
	Single compartment dose (Gy)	34.9	63.7	45	45.2	62.2	–	–
	Dosimetry method	PET/CT	PET/CT	PET/CT	PET/CT	PET/CT	MicroCT	MicroCT
Mass	Mean dose (Gy)	45.5	57.6	58.1	45.4	64.1	76.7	11.3
	D10 (Gy)	95.2	122.9	91.5	94.8	137.0	131.4	19.3
	D30 (Gy)	58.6	70.2	70.8	54.9	80.0	80.8	11.9
	D50 (Gy)	35.7	37.6	55.8	36.1	52.6	59.4	8.73
	D70 (Gy)	22.6	20.2	41.6	24.2	27.9	44.8	6.58
	D90 (Gy)	8.5	10.0	26.9	8.1	7.2	30.6	4.49
	V30	58.3	55.9	85.4	60.4	68.9	90.6	3.28
	V50	37.2	40.6	57.8	34.5	52.6	62.4	0.96
	V60	28.9	34.9	44.0	26.8	43.5	49.3	0.64
	V80	16.5	26.3	20.3	15.8	30.0	30.6	0.34
	V100	9.1	19.0	6.4	8.4	19.3	10.3	0.22
Hemisphere	Mean dose (Gy)	33.8	65.3	41.1	45.0	61.4	41.4	6.08
	V20	54.8	74.6	68.2	65.5	77.5	63.1	3.39
	V30	40.8	66.4	57.6	50.8	67.1	49.8	1.21
	V50	23.0	52.2	37.1	33.6	49.8	28.8	0.39
	V60	17.3	44.7	27.7	27.3	41.7	21.5	0.27
Cortex	Mean dose (Gy)	15.4	27.6	19.2	16.7	33.3	–	–
	V20	24.1	33.3	32.6	24.1	43.8	–	–
	V30	17.7	28.2	25.6	17.7	36.5	–	–
	V50	10.1	21.3	15.3	10.7	25.5	–	–
	V60	7.4	18.0	11.3	8.3	21.0	–	–
	T:N^‡^	2.96	2.08	3.02	2.71	1.93	–	–

^*^P5 expired 15 h post-treatment. The “Actual” column lists the absorbed doses delivered at the time of expiration. P5 “Extended” represents the absorbed doses that would have been delivered had the dog survived

^†^Determined from post-mortem histopathology, in all other animals, the diagnosis is based on pre-treatment imaging

^‡^T:N has been calculated as the ratio of the absorbed dose to the tumor compared to normal cortical brain tissue

### Post-procedural care

All animals received a neurologic exam daily for at least the first 2 days post-therapy with additional exams scheduled weekly until neurologically normal and at return imaging visits (Fig. 1). Oral prednisone (1 mg/kg/day) was started on day 1 in all animals for neuroinflammatory prophylaxis and tapered at the discretion of the study veterinarian. Research dogs were survived for 1 month, and after euthanasia, the brains were extracted for histopathologic analysis and microCT scan for microdosimetric analysis. Patient dogs received post-treatment MRI at 1, 3, and 6 months after treatment and were followed bi-monthly with their owner to assess long-term progress until death.

### Explanted brain microdosimetric analysis

Scanning of formalin-fixed, whole brain hemispheres in R1–R3, P2, and P5 was performed on a Bruker Skyscan 1176 micro-CT (Bruker Corporation, Kontich, Belgium) with a 68-mm axial field of view (FOV) and 8.74-μm isotropic voxels. The following technical parameters were utilized: 656 projections, 90 kVp, 0.695–1.390 mAs, Cu+Al beam filtration, and an 8.74-μm pixel size. The brain hemispheres were carefully wrapped in parafilm prior to the scan to prevent desiccation and movement of the spheres during acquisition. MicroCT-scanned tissue was analyzed using an algorithm that can quantitatively identify 3D locations of individual glass ^90^Y microspheres [21]. Microscopic 3D radiation absorbed dose maps were created by convolving a dose point kernel (DPK) of a single glass microsphere with the microsphere locations determined using the microsphere identification algorithm. The DPK was calculated for 36-μm voxels using MCNP 6.0 (Los Alamos National Laboratory, Los Alamos, NM) with the ^90^Y beta spectrum defined by Eckerman [22] in an 5 × 5 × 5 cm block of brain tissue [23]. Electron/photon-coupled transport simulation of primary and secondary particles was performed with an energy cutoff of 1 keV. GPU-accelerated convolution in the frequency domain with the fast Fourier transform was used to decrease computation time. Finalized absorbed dose maps had a voxel size of 36 μm. All microdosimetry was performed using MATLAB R2018B. Additional details related to this process are described in Pasciak et al. [18]. This microscopic information allowed absorbed dose analysis with much greater accuracy and fidelity than PET/CT-based dosimetry, without degradation from limited resolution or partial volume effect.

In patient dogs, dose metrics such as D70 (minimum dose to 70% of mass volume) and the V60 (percent mass volume receiving more than 60 Gy) were computed. Dose-volume histograms were also computed for normal and abnormal tissue structures. Tissue structures of interest were manually contoured on microCT based on orthogonal comparison to images acquired during gross dissection and MRI. In research animals and deceased patients, these data were calculated based on microCT-based analysis, while in living patient animals, quantitative ^90^Y PET/CT [19] was used.

### Histopathologic analysis

After hemispherical microCT, weights of treated and untreated hemispheres were obtained and gross dissection of R1–R3 was performed. The midbrain was transected to remove the brainstem and cerebellum from the remainder of the brain. Coronal sections approximately 3–5 mm in thickness were made of the cortex and subcortical gray matter structures. Axial sections approximately 3–5 mm in thickness were made of the brainstem and cerebellum. A complete gross examination was performed, noting any areas of suspected infarction/necrosis. The tissue was then paraffin-embedded for microscopic sectioning and staining. Hematoxylin and eosin-stained slides were analyzed via light microscopy by human (MM) and veterinary (CZ) neuropathologists. Areas of infarct or encephalomalacia, bead distribution, and inflammatory processes, as well as any other abnormalities, were noted and classified as ante-, peri-, or postmortem. Gross analysis and histopathology for P2 and P5 were performed using the same methodology as described above with analysis by veterinary pathologists (CB, CZ).

## Results

**Fig. 3:**
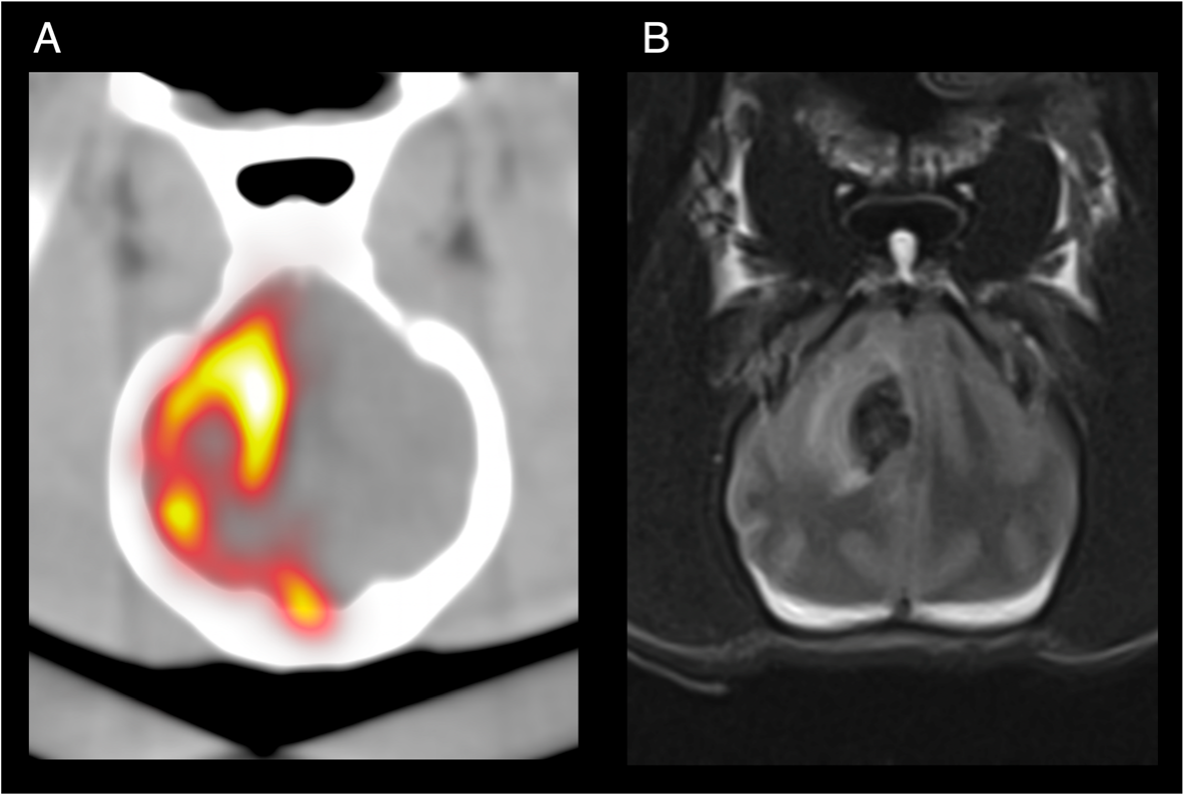
**a** Post-treatment ^90^Y PET/CT demonstrating technical success in P1. **b** Pre-treatment MRI obtained 16 days prior to therapy

**Fig. 4:**
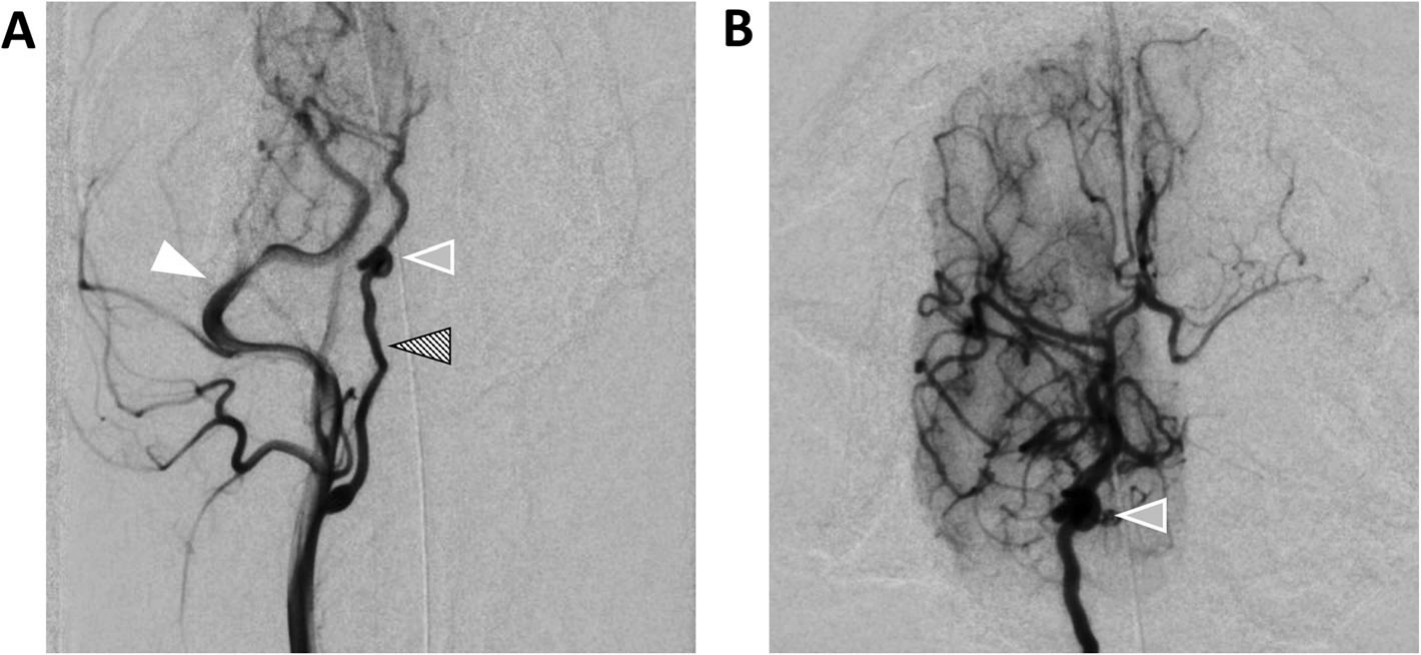
Angiography of P1 prior to infusion of ^90^Y-RE. **a** Common carotid artery angiogram detailing the dominant external carotid artery (solid white arrow), extracranial ICA (crosshatched arrow), and tortuosity of the ICA as it enters the skull base (gray arrow). **b** ICA angiogram

Cerebral ^90^Y-RE was performed in all research (R1–R3) and patient (P1–P5) dogs with a qualitative agreement between expected treated territory and post-treatment ^90^Y PET/CT (R1–R3) or the target lesion on pre-treatment MRI and ^90^Y PET/CT (P1–P5). Figure 3 compares post-treatment ^90^Y PET/CT and pre-treatment MRI in P1. As patient dog lesions were perfused primarily from the MCA, infusion of spheres was performed with the microcatheter tip in the ICA proximal to the cavernous segment. Because of the tight tortuosity of the canine cavernous carotid, further subselection was not possible (Fig. 4a, b, gray arrow). Treatment efficiencies and delivery rate are provided in the Supplemental Material.

### Treatment efficiencies and delivery rates

Owing to the small lumen of the Excelsior SL-10 microcatheter (0.021”, 1.7 Fr), the 20-mL/min minimum infusion rate recommended for the TheraSphere delivery system could not be achieved. An average of 7.2 [6.6–9.2] mL/min across 8 treatments was achieved with a delivery efficiency ranging from 85 to 98% of the prescribed activity. All animals (research and patient) were successfully recovered from anesthesia following the procedure.

### Research animal dosimetry and clinical course

Based on 3D volumetry from cone beam CT and the administered activity, the average absorbed dose to treated tissue using the MIRD method [20] in R1–R3 was 27.9, 66.5, and 73.0 Gy, respectively.

All research animals had an unremarkable neurological exam prior to treatment. R1 exhibited apathetic behavior, depressed left menace response, and left front hemiwalking at 2 days post-treatment. In R1, these findings were self-limited and resolved 7 days post-treatment. Following treatment, R2 exhibited circling to the left, delayed right hemiwalking, delayed right front and rear proprioception, decreased right facial sensation, and increased right patellar reflex at 2 days post-therapy. Once again, these symptoms were self-limited, and R2 had a normal neurological exam at 12 days post-treatment. In R3, neurological exam was within normal limits at 2 days post-treatment. After behavior and neurologic symptoms normalized in R1–R3 shortly after treatment, these animals remained normal and healthy for the duration of their survival.

**Fig. 5:**
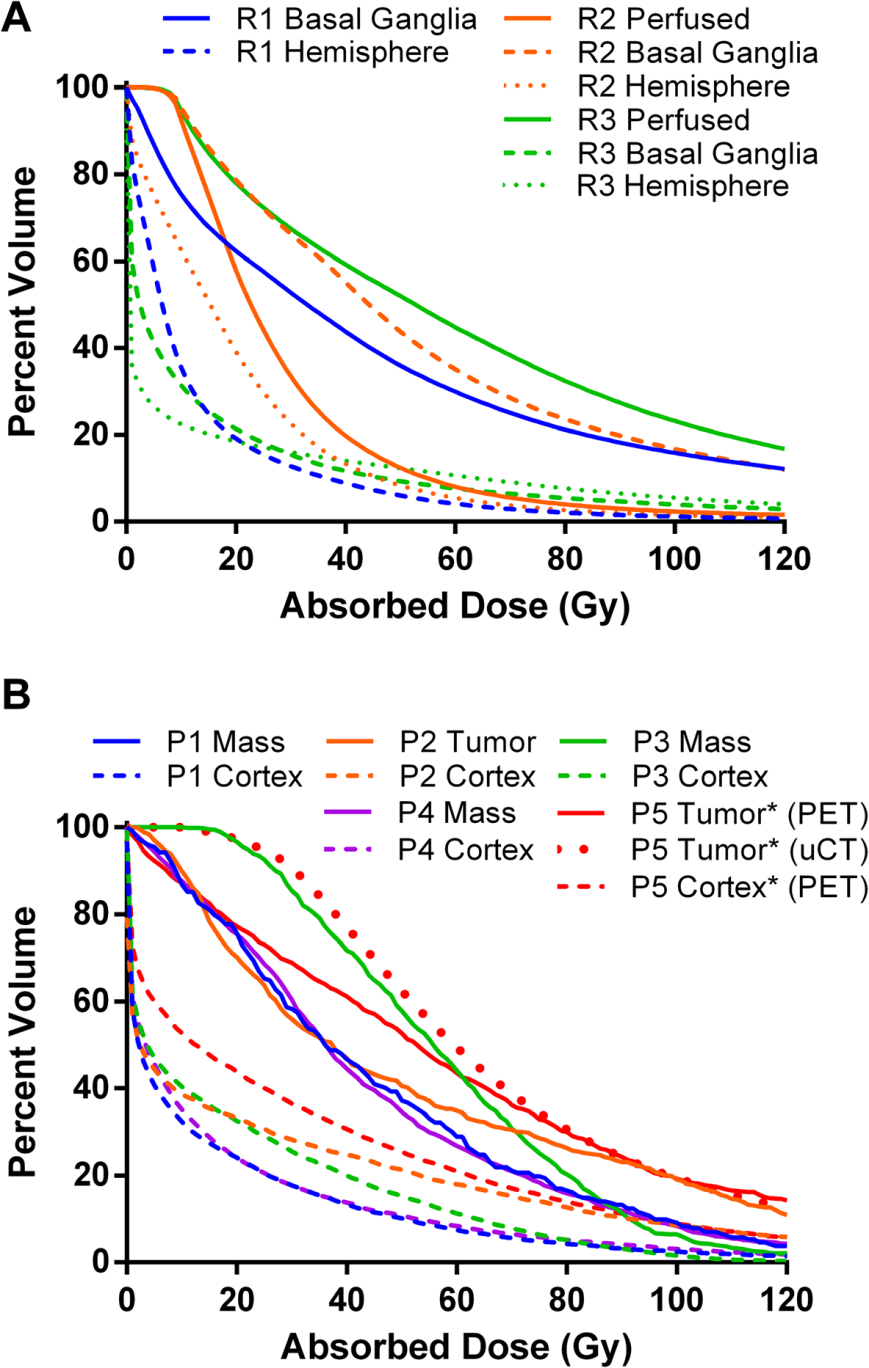
Dose-volume histograms. **a** R1-R3 based on microCT identification of microspheres in the treated hemisphere after euthanasia and organ extraction. **b** P1–P5 based on post-infusion quantitative ^90^Y PET/CT. MicroCT-based dose-volume histogram is also provided for P5 post-mortem. *P5 values are extrapolated assuming this animal had survived. For actual dose delivered, see Table 2

Based on a fully 3D microdosimetric analysis of the explant-treated hemisphere and identification of individual microspheres, the average absorbed dose to the treated territory in R1–R3 was 14.0, 30.9, and 73.2 Gy, respectively. Maximum hemisphere absorbed doses exceeded 1000 Gy in all animals, and absorbed doses to the thalamus and basal ganglia were 55.9, 63.4, and 18.3 Gy in R1–R3, respectively. Also, of interest is the thalamic V100, which was 15.8 and 16.7% respectively for R1 and R2 but only 3.9% in R3. Additional dosimetric and treatment-related details for R1–R3 are listed in Fig. 5a and Table 1.

### Research animal histopathology R1–R3

Gross examination of whole brains in R1–R3 showed no evidence of volume asymmetry or atrophy. The difference in weight between the two hemispheres for each research dog was minimal at less than 1% (range of 0.28–0.43 g).

Histologic analysis from 20 tissue blocks from the treated hemisphere in each animal (R1–R3) demonstrated 9, 5, and 20 foci consistent with chronic infarcts ≤ 5 mm in size, with the vast majority of infarcts measuring less than 2 mm. The infarcts most commonly involved the cortical gray matter and gray-white matter junction and often were associated with microspheres, suggesting the infarcts may be from microspheres occluding small vessels. R1–R3 also exhibited a bilateral distribution of perivascular chronic inflammation and, in R1 and R3, a similarly broad distribution of granulomatous inflammation. Neither the perivascular inflammation nor the granulomatous inflammation showed any significant association with the microspheres. GFAP and Iba1 immunostainings highlighted diffuse microglial activation and reactive astrocytosis. GMS, AFB, and Gram staining showed no evidence of infectious organisms. These findings were determined to be the result of a known issue with hydrophilic coatings on the catheters used [24, 25], and likely unrelated to the microspheres themselves.

### Patient animal dosimetry

The MIRD absorbed doses were 34.9, 63.7, 45.0, 45.2, and 62.2 Gy to the treated hemisphere in P1–P5, respectively. Through quantitative analysis of post-treatment ^90^Y PET/CT, average hemisphere absorbed doses were 33.8, 65.3, 41.1, 45.0, and 9.2 Gy in P1–P5, respectively. As discussed below, P5 expired 15 h after treatment resulting in an actual absorbed dose delivery at the time of death of 9.2 Gy. Based on post-treatment ^90^Y PET/CT, if P5 had survived, the extended absorbed dose delivery to the treated hemisphere would have been 61.4 Gy (Table 2). Absorbed doses to the mass in P1–P5 were 45.5, 57.6, 58.1, 45.4 Gy, and 64.1 Gy (extended, P5) with a V60 ranging from 26.8 to 44.0% (Table 2, Fig. 5b). Mean cortical doses (exclusive of mass) were 15.4, 27.6, 19.2, 16.7, and 33.3 Gy with a V60 averaging 13.2% (range 7.4–21.0%) to the cortex across all animals. The tumor-to-normal uptake ratio (T:N) for unilateral ICA infusion of ^90^Y-RE ranged from 1.93 to 3.02 (Table 2). Extended absorbed doses calculated by individual identification of microspheres demonstrate preferable tumor dosimetry compared to PET/CT-based calculations with a mean tumor dose of 76.7 vs 64.1 Gy. Differences between the microCT-based and PET-based dosimetry are also apparent when comparing the differential dose metrics (Table 2) or DVH (Fig. 5b). For example, V30 for tumor was 90.6% based on microCT and 68.9% based on PET/CT—differences which are likely a function of resolution limitations of PET/CT and/or partial volume effects in the setting of small lesions. Because all of the lesions in P1–P5 were small, lesion absorbed doses based on ^90^Y PET/CT (Table 1) are likely underestimated.

### Patient animal pre-treatment clinical presentation

Prior to treatment, all patient animals initially presented with generalized seizure activity. Neurological exam at the presentation on the day of treatment demonstrated no abnormalities in P2–P4, although P2 experienced a generalized seizure immediately prior to anesthesia induction. P1 showed a preference for turning to the right vs left but was otherwise normal. P5, however, displayed clear neurologic defects on the day of treatment including left side proprioceptive defects and a left visual deficit and possible circling to the right.

Table 3 describes the presenting symptoms of P1–P5, as well as relevant medications used prior to treatment in an attempt to control symptoms. The baseline symptoms are with the medical management listed.

**Table 3:** Summary of pre-treatment imaging diagnosis, baseline symptoms, and pre-treatment medical management in P1–P5

Patient	Official diagnostic basis for inclusion	Baseline symptoms	Medical management at seizure onset
P1	Highly vascular neoplasm with a component of hemorrhage vs vascular anomaly	First seizure 1 month prior to ^90^Y-RE with compulsive circling to the right. At presentation prior to ^90^Y-RE, there was a preference for turning to the right vs left. Remainder of neurological exam was normal.	Phenobarbital, low-dose prednisone
P2	Astrocytoma*	First seizure 3 months prior to ^90^Y-RE. No neurological deficits on presentation prior to treatment.	Levetiracetam
P3	Low-grade glioma	First seizure 3 months prior to ^90^Y-RE. No neurological deficits on presentation prior to treatment.	Levetiracetam, low-dose prednisone
P4	Glial neoplasm	First seizure 2 months prior to ^90^Y-RE. At presentation prior to ^90^Y-RE, there was a left hindleg slight proprioceptive delay.	Levetiracetam, gabapentin, low-dose prednisone
P5	Astrocytoma*	First seizure 1 month prior to ^90^Y-RE. At presentation prior to ^90^Y-RE, there were left side proprioceptive defects and left visual deficit and circling to the right.	Levetiracetam, low-dose prednisone

*Proven from post-mortem pathology

### Patient animal post-treatment clinical findings

P1–P3 displayed focal cerebral cortical deficits at 1 day post-treatment, including circling to the side of the lesion and contralateral visual deficit, proprioceptive delay, and weakness (P1–P2 only). In P1, these findings were present prior to treatment. On post-treatment days 33, 20, and 29 in dogs P1–P3, respectively, neurological exams normalized (Fig. 1); however, P1 continued a preference for turning to the right and circling to the right while under stress. P4 demonstrated no post-procedural deficits. After treatment, P5 was able to walk and eat; however, he subsequently had a seizure several hours later and expired 15 h post-treatment from ventilatory arrest. A multidisciplinary investigation determined that the cause of death may be due to patient selection, treatment side effect, or periprocedural complications. While the cause has not been definitively determined, M&M findings are listed in the supplement.

P1–P4 demonstrated improvement in clinical status by their 1 month follow-up visit, with no new seizures or neurologic deficits (Fig. 1). Steroids were discontinued in all animals within 33 days of treatment. P1 continued to perform well with normal neurologic exams at 3- and 6-month visits and no seizures and is currently asymptomatic 12 months post-treatment other than the stable circling under stress noted above. P3 and P4 have subjectively normalized behavior compared to pre-treatment and continue to have normal neurologic exams. P3 has had no seizures up to 8 months post-therapy. P2 was restarted on phenobarbital due to recurrence of seizure activity at 53 days post-treatment; however, drowsiness precluded reaching therapeutic dose, and the animal had additional seizures on days 95, 122, and 143 post-treatment. P2 was ultimately euthanized on day 154 per owner request due to documented expansion of the intra-axial mass on MRI (Fig. 2b). P4 had a few isolated seizures at 4 months, and phenobarbital was added with an isolated recurrence of a single short seizure shortly after the 6-month MRI wherein it was determined that phenobarbital levels were at the low end of the therapeutic range.

### Patient animal post-treatment radiologic findings

**Fig. 6:**
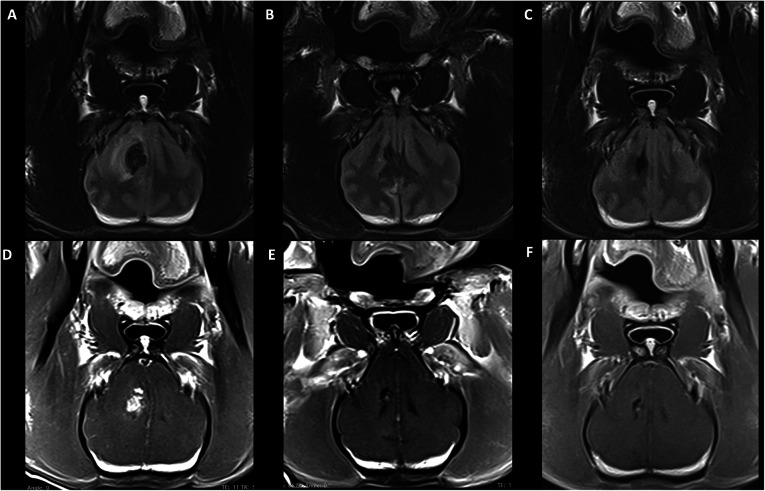
**a**–**c** P1 T2-FLAIR imaging at 2 weeks prior to treatment (**a**), 1 month (**b**), and 6 months (**c**) following ^90^Y-RE. **d**–**f** T1 post-contrast imaging at 2 weeks prior to treatment (**d**), 1 month (**e**), and 6 months (**f**) following ^90^Y-RE. At 1 month post-therapy, there is a decrease in lesion size with a resolution of perilesional edema and absence of contrast enhancement. The resolution of midline shift without evidence of cortical atrophy was also noted. Treatment effect is maintained at 6 months with a continued decrease in lesion size. Additional images showing the progression of P4 which had a less impressive response are shown in the supplement

At 1 month post-therapy, MRI of P1 showed a 94% reduction in mass size, resolution of perilesional edema, and falx shift as well as the absence of contrast enhancement. Three- and 6-month post-treatment MRI showed further reduction of mass size and continued absence of contrast enhancement (Fig. 6). At 1 month post-therapy, P2 demonstrated a 47% reduction in mass volume also with a resolution of falx shift and perilesional edema. The lesion, however, had returned to the initial pre-treatment size at 3 months. P3 and P4 demonstrated decreased mass volume on T2 FLAIR at 1, 3, and 6 months post-treatment; however, at 6 months, the mass volume of P4 was nearly back to the pre-treatment size with new T1 post-contrast enhancing component (Fig. 2b).

**Fig. 7:**
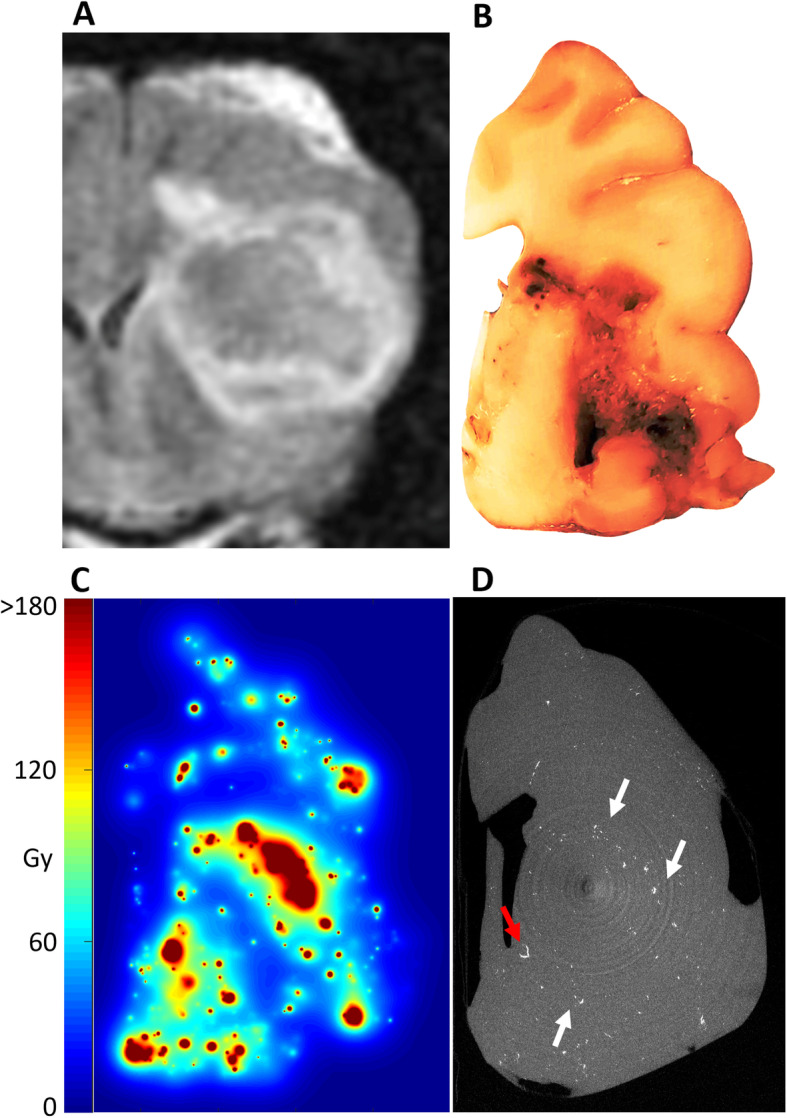
Gross pathology and microdosimetry for P5. **a** Pre-treatment T2 FLAIR 1 month prior to therapy. **b** Formalin-fixed gross pathologic example of the involved hemisphere with significant central tumor necrosis. **c** Microdosimetry showing the absorbed dose distribution in dog P5 if the dog had survived. At the time of death, only 15% of the absorbed doses shown had been delivered based on the half-life of ^90^Y. **d** Post-explant microCT showing the gross distribution of glass microspheres. the image is a maximum intensity projection of 100 microCT slices with a combined thickness of 900 μm. A preference for deposition in the peri-necrotic region of the tumor can be seen (white arrows). Occasional filling of end arterioles/capillaries with microspheres can be visualized (red arrow)

Cortical atrophy was not appreciated in post-treatment imaging (1–6 months) in any patient animal. Quantitative analysis of mass volumes as a function of time post-treatment is shown in Fig. 2b. While Fig. 6 describes the radiologic course of P1 following treatment, this animal had the best response of all treated patient animals with intra-axial masses. Figure 7 shows the radiologic progression of P4 at 1 month prior to treatment, 1 month post-treatment, and 6 months post-treatment. While tumor volume on T2 FLAIR was decreased at 1, 3, and 6 months (Fig. 7), the progression shown in the images below is less dramatic than P1. P1 was the only animal to reach 1-year post-treatment survival at the time of this publication, and the 12-month MRI showed magnetic susceptibility artifact of the right frontal lobe, high T2 signal structural change of the right parietal lobe suggestive of encephalomalacia, and overall progressive structural changes consistent with brain atrophy. The lesion at 12 months showed stable radiologic response compared to 3- and 6-month imaging, and the animal continues to be clinically asymptomatic. A future publication hopes to detail long-term radiologic and clinical findings associated with all treated animals at more than 12 months post-therapy.

### P2 and P5 histopathologic findings

In P2, imaging findings were consistent with progressive ipsilateral transtentorial herniation but did not show chronic signs of transtentorial herniation on histopathologic review. There were imaging findings of transtentoral herniation in P5 as well prior to therapy. Although signs of chronic herniation were not seen on gross or histological pathological analysis, there was gross asymmetry of the brain with the right hemisphere being larger anteriorly after fixation. Both P2 and P5 histologic tumor characteristics were consistent with astrocytic neoplasm. In the analysis of P2 at 5 months post-treatment, there was multifocally extensive necrosis in the tumor as well as areas of viable tumor some with aggressive/invasive extension into ventricles, meninges, and along vasculature. In P5, scattered foci < 2 mm of edema and rarefaction were noted, as well as scattered laminated thrombotic small vessels, both of which were sometimes associated with microspheres. Multifocal perimortem petechial hemorrhage was also identified and was distributed in areas remote from the tumor, including the cerebellum and brainstem. These findings were determined to most likely due to perimortem manipulation and attempted therapies, given their acute appearance and lack of association with microspheres. No other acute histologic abnormalities and no evidence of focal intracranial bleed were noted.

## Discussion

Cerebral ^90^Y-RE was technically feasible in both healthy dogs and in dogs with presumed spontaneous brain cancers. Preliminary efficacy results are encouraging with a potential increase in mean survival time compared historically to dogs that undergo medical management alone [26]. Safety is a concern given the embolic nature of RE, particularly since all treatments of mass-bearing animals were performed from the ICA, treating at least half the brain. However, while 2 of 3 research dogs and 4 of 5 patient dogs exhibited acute post-treatment neurological changes, these findings were transient and quickly declined in severity with resolution 7–28 days post-therapy. Importantly, despite hemispheric delivery of ^90^Y in patient dogs, all animals except for P2 received a higher absorbed dose to the mass compared to uninvolved cortical matter in the treated hemisphere. T:N ranged from 1.93 to 3.02, and absorbed doses to the intra-axial mass in P1–P5 [45.4–75.7 Gy] were comparable to the 60 Gy standard of care in EBRT. However, this should be interpreted in the context that the tissue-dependent biological effect of ^90^Y-RE may be reduced compared to EBRT owing to the absorbed dose non-uniformity at the microscopic scale. Additional data is needed to characterize this difference in both CNS tissue and CNS tumors.

At the time of treatment, diagnosis in P1–P5 was based on clinical symptomatology and an imaging appearance suggestive of cancer (Table 2), although no diagnoses were biopsy-proven. P1 and P3 are currently asymptomatic at 370 and 272 days post-diagnosis while P4 is normal at 194 days aside from occasional seizures due to low phenobarbital levels. P2 survived 191 days—also substantially longer than expected with medical management alone [26]. Under the caveat that this is a small pilot study without biopsy-proven diagnoses, radiographic-demonstrated control of the intra-axial mass in P1–P4 at 1 month post-therapy with continued disease-free survival in P1 and radiographically improved disease with no focal neurologic exam deficits in P3 and P4 is encouraging.

Reviewing the absorbed doses from R1–R3 and associated neurologic deficits, it is interesting that R3 had the highest absorbed dose (73.0 Gy), but had no neurologic sequelae. A possible explanation is, due to differences in catheter position at the time of infusion (ICA, MCA, PCA), absorbed doses to the thalamus and basal ganglia were 55.9, 63.4, and 18.3 Gy in R1–R3. The thalamic V100 was 15.8 and 16.7% respectively for R1 and R2 but only 3.9% in R3. The higher thalamic and basal ganglia absorbed dose and corresponding microsphere concentration may explain the transient post-treatment neurological exam findings in R1 and R2 which were absent in R3 (Fig. 5a).

Whole-brain radiation using EBRT is common clinical practice for tumor control in cases of multifocal metastatic disease [9], and while this may seem superficially similar to the hemispheric treatments performed in this investigation, there is one key difference. ^90^Y-RE has two potential modes of toxicity to normal brain tissue: (1) radiation toxicity and (2) ischemic toxicity. Given the half-life of ^90^Y, the radiation emitted is a protracted exposure, with about 90% of the radiation dose deposited in the first 9 days after therapy. On the first day post-treatment, only a small fraction of the target radiation dose has been delivered. Therefore, it is likely that the acute findings in R1, R2, and P1–P3 were the result of the ischemic change from infused microspheres which was identified histologically in all research animals. Due to the high radioactivity per sphere of glass microspheres, the total volume of embolic used in these animals was small (0.00066–0.0021 mL, Tables 1 and 2). However, restricting future treatments to days 2–5 post-calibration has the potential to reduce the embolic burden. This discussion should be considered in the context that super-selective angiography is routine in human neurointervention [27–29] and would likely further lessen toxicity should cerebral ^90^Y-RE be translated to humans.

**Fig. 8:**
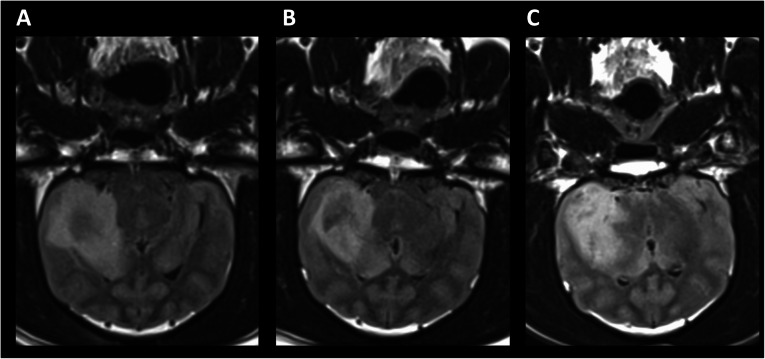
P4 T2 FLAIR at **a** 1 month pre-treatment, **b** 1 month post-treatment, and **c** 6 months post-treatment

^90^Y has a heterogeneous microscopic absorbed dose distribution when administered in the liver. In the liver, the greatly decreased toxicity of ^90^Y-RE compared to EBRT has been attributed to this micro-scale heterogeneity [18, 30, 31]. A similar pattern of microscopic absorbed dose heterogeneity has been found in the normal cortical brain tissue within the treatment territory (Fig. 8). Whether this phenomenon leads to decreased neurotoxicity is to be determined. However, in many ways, this microscopic heterogeneity may recapitulate the normal tissue sparing effects seen in microchannel proton and X-ray microbeam radiation therapy, which are novel areas of ongoing EBRT research [32–34].

This study has several limitations. First, the study has both a limited number of research and patient dogs. In research animals, the 1-month follow-up may be insufficient to fully characterize clinical toxicity and/or histologic findings. Similarly, follow-up was limited in patient animals to 6 months post-therapy without histologic analysis in surviving animals. Second, pre-treatment diagnoses in P1–P5 were based on imaging appearance only, and the histopathologic diagnosis was confirmed only in P2 and P5. The lack of confirmed pre-treatment diagnosis may result in variable radiosensitivity among treated tumors and may affect the observed efficacy. Third, the reported PET/CT-based dose metrics (Table 2, Fig. 5b) should be interpreted in the context that all results may be underestimated for small masses (2.7–8.6 mL in P1–P5) due to the resolution limitations of ^90^Y PET/CT [35]. The dosimetry based on microCT (R1–R3, P5) should, however, be accurate. Fourth, due to the acute angle, the ICA enters the skull base in dogs (Fig. 3), primary cerebral arteries were not accessible from an ICA approach, and patient dog ^90^Y-RE deliveries were made from the extracranial ICA. Finally, as mentioned above, animals in this study were treated at different time points post-calibration of ^90^Y glass microspheres resulting in a different microsphere-specific activity which may introduce yet another variable in the observed dose-response relationship.

^90^Y-RE is technically feasible in both healthy canine models and canine patients with presumed intra-axial cancers. Delivery of absorbed doses comparable to EBRT to intra-axial masses was achieved with average cortical parenchymal doses that were ~ 2–3 times lower. Acute post-treatment neurologic abnormalities were identified in 6 of 8 animals; however, these effects were transient. In future attempts, acute post-treatment neurologic abnormalities may be reduced with selective ^90^Y-RE delivery and with a treatment day closer to the microsphere calibration date. While preliminary, initial data on the efficacy of ^90^Y-RE as a potential treatment for brain cancer is encouraging, however, additional investigation is needed to further evaluate both safety and efficacy.

## Supplementary information

**Additional file 1:.** M&M findings on P5.

## Data Availability

The datasets used and/or analyzed during the current study are available from the corresponding author on reasonable request.

## Abbreviations

RE: Radioembolization
^90^Y: Yttrium-90
EBRT: External beam radiation therapy
V60: The percent volume of tissue receiving more than 60 Gy
D70: The lowest absorbed dose to 70% of the volume
MIRD: Medical internal radiation dose
T:N: Tumor to normal uptake ratio
Hemiwalking: Forcing the animal to hop on the front and rear limb on a single side to test postural reaction and proprioception
Menace response: Blinking in response to a rapidly approaching object to test cranial nerves and vision
ICA: Internal carotid artery
PCA: Posterior cerebral artery
MCA: Middle cerebral artery
