# Correction to: Yttrium-90 radioembolization as a possible new treatment for brain cancer: proof of concept and safety analysis in a canine model

**DOI:** 10.1186/s13550-020-00691-5

**Published:** 2020-09-10

**Authors:** Alexander S. Pasciak, Sasicha Manupipatpong, Ferdinand K. Hui, Larry Gainsburg, Rebecca Krimins, M. Christine Zink, Cory F. Brayton, Meaghan Morris, Jaime Sage, Danielle R. Donahue, Matthew R. Dreher, Dara L. Kraitchman, Clifford R. Weiss

**Affiliations:** 1 School of Medicine, The Johns Hopkins University School of Medicine, 1800 Orleans St, Baltimore, MD 21287 USA; 2 Department of Radiology and Radiological Science, Division of Vascular and Interventional Radiology, The Johns Hopkins University School of Medicine, Baltimore, MD USA; 3 Mid-Atlantic Veterinary Neurology and Neurosurgery, Baltimore, MD USA; 4 Department of Molecular and Comparative Pathobiology, The Johns Hopkins University, Baltimore, MD USA; 5 Department of Radiology and Radiological Science, Express Radiology Research Lab, The Johns Hopkins University School of Medicine, Baltimore, MD USA; 6 Department of Radiology and Radiological Science, Veterinary Clinical Trials Network, The Johns Hopkins University School of Medicine, Baltimore, MD USA; 7 Department of Anesthesiology and Critical Care Medicine, The Johns Hopkins University School of Medicine, Baltimore, MD USA; 8 Department of Pathology, The Johns Hopkins University School of Medicine, Baltimore, MD USA; 9 MRI Vets, PLLC, Georgetown, TX USA; 10 Mouse Imaging Facility, National Institutes of Health, Bethesda, MD USA; 11 Biocompatibles UK Ltd., a BTG International group company, Farnham, Surrey UK; 12 Department of Radiology and Radiological Science, Center for Image-Guided Animal Therapy, The Johns Hopkins University School of Medicine, Baltimore, MD USA; 13 Department Biomedical Engineering, The Johns Hopkins Whiting School of Engineering, Baltimore, MD USA

**Correction to: EJNMMI Res 10, 96 (2020)**

**https://doi.org/10.1186/s13550-020-00679-1**

Following publication of the original article [1], the authors reported that the captions of Figs. 7 and 8 had been erroneously swapped in the article.

**Fig. 7:**
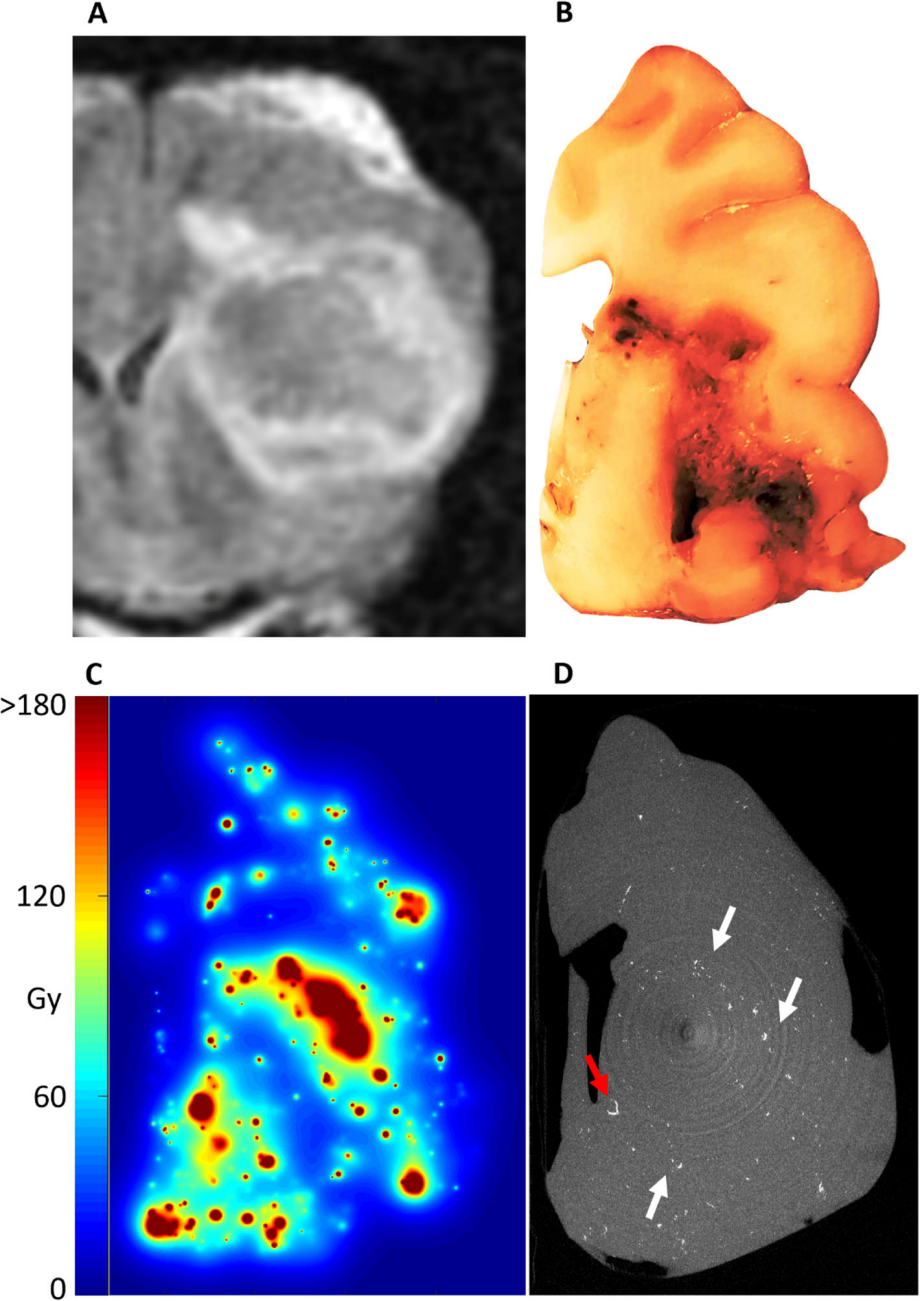
Gross pathology and microdosimetry for P5. **a** Pre-treatment T2 FLAIR 1 month prior to therapy. **b** Formalin-fixed gross pathologic example of involved hemisphere with significant central tumor necrosis. **c** Microdosimetry showing the absorbed-dose distribution in dog P5 if the dog had survived. At the time of death, only 15% of the absorbed-doses shown had been delivered based on the half-life of ^90^Y. **d** Post-explant microCT showing gross distribution of glass microspheres. Image is a maximum intensity projection of 100 microCT slices with a combined thickness of 900 um. A preference for deposition in the peri-necrotic region of tumor can be seen (white arrows). Occasional filling of end-arterioles/capillaries with microspheres can be visualized (red arrow)

**Fig. 8:**
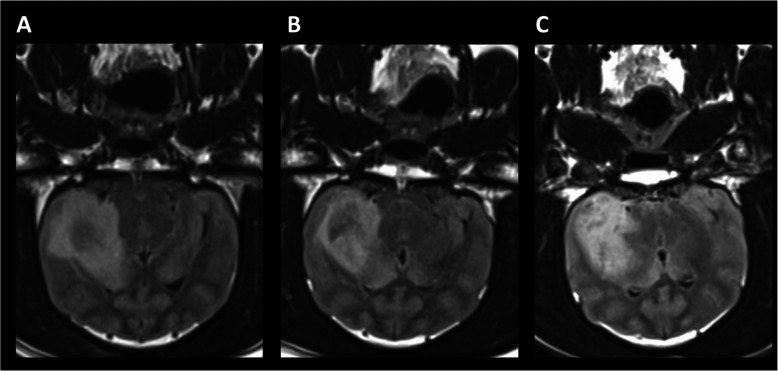
P4 T2 FLAIR at **a** 1 month pre-treatment, **b** 1 month post-treatment and **c** 6 months post-treatment

The figures have now been corrected in the published original article.

In addition, please find the (corrected) figures in this correction for reference.
