# Supplementary material for: Yttrium-90 radioembolization as a possible new treatment for brain cancer: proof of concept and safety analysis in a canine model
Source: EJNMMI Res. 2020 Aug 17;10:96. doi: 10.1186/s13550-020-00679-1 (PMC7431501; doi:10.1186/s13550-020-00679-1)
Supplement: Supplementary file 1 — Additional file 1:. M&M findings on P5. [file 13550_2020_679_MOESM1_ESM.docx]

**Supplemental Materials**

*M&M findings on P5*

A multidisciplinary investigation into the cause of death with outside veterinary neuropathology, neurosurgery, radiology, and anesthesiology consultation was conducted. There were multiple factors that may have contributed to the animal’s death. The investigation was unable to find a definitive cause, and the true cause may be a combination of findings listed below. Possible factors contributing to death identified by the M&M were divided into four categories: 1) patient selection, 2) intraprocedural, 3) post-procedural and 4) ischemia-related issues. 1) From a patient selection standpoint, this patient had a large, hypervascular brain tumor with a volume on T2 FLAIR and T1 post-contrast equivalent to 18% and 12% of cerebral volume, respectively, which may have put the animal at increased risk for complication. In addition, the pre-treatment MRI was remote, occurring >30 days prior to therapy. Post-treatment review of the MRI images revealed evidence of transtentorial herniation. Although signs of chronic herniation were not seen on gross or histological pathological analysis, there was gross asymmetry of the brain with the right hemisphere being larger anteriorly after fixation. Existing herniation prior to therapy would greatly increase the risk of complication. 2) Intraprocedural issues which may have contributed to the cause of death include a lengthy angiography time from bilateral vascular mapping which when coupled with a high maintenance IV fluid rate may have led to overhydration. High-dose IV dexamethasone (0.1 mg/kg) was not given to P5 because the animal was already on oral prednisone; however, this dosage is less than what would have been given for cerebral edema Furthermore, during the post-treatment PET/CT, the animal was found to be hyperthermic and tachypneic with a high heart rate on recovery, all of which can contribute to cerebral edema. 3) Post-treatment, while the dog was initially responsive, a seizure occurred ~4.5 hrs after recovery. At that time, the dog was treated with intravenous midazolam and mannitol, but may have benefited at that time from more aggressive repeated interventions for cerebral edema and compromised respiration (i.e., mannitol, hypertonic saline, intubation with hyperventilation). 4) Finally, ischemia and edema related to angiography and/or embolization could have played a role in the animal’s decompensation; however, there was no evidence of widespread ischemia found on histopathological analysis.
